# DNA barcoding analysis and phylogenetic relationships of tree species in tropical cloud forests

**DOI:** 10.1038/s41598-017-13057-0

**Published:** 2017-10-02

**Authors:** Yong Kang, Zhiyan Deng, Runguo Zang, Wenxing Long

**Affiliations:** 1 Hainan Key Laboratory for Sustainable Utilization of Tropical Bioresource; Institute of Tropical Agriculture and Forestry, Hainan University, Haikou, 570228 China; 2 Key Laboratory of Forest Ecology and Environment of State Forestry Administration; Institute of Forest Ecology, Environment and Protection, Chinese Academy of Forestry, Beijing, 100091 China

## Abstract

DNA barcoding is a useful tool for species identification and phylogenetic construction. But present studies have far reached a consistent result on the universality of DNA barcoding. We tested the universality of tree species DNA barcodes including *rbcL*, *matK*, *trnH*-*psbA* and ITS, and examined their abilities of species identification and phylogenetic construction in three tropical cloud forests. Results showed that the success rates of PCR amplification of *rbcL*, *matK*, *trnH*-*psbA* and ITS were 75.26% ± 3.65%, 57.24% ± 4.42%, 79.28% ± 7.08%, 50.31% ± 6.64%, and the rates of DNA sequencing were 63.84% ± 4.32%, 50.82% ± 4.36%, 72.87% ± 11.37%, 45.15% ± 8.91% respectively, suggesting that both *rbcL* and *trnH*-*psbA* are universal for tree species in the tropical cloud forests. The success rates of species identification of the four fragments were higher than 41.00% (*rbcL*: 41.50% ± 2.81%, *matK:* 42.88% ± 2.59%, *trnH-psbA:* 46.16% ± 5.11% and ITS: 47.20% ± 5.76%), demonstrating that these fragments have potentiality in species identification. When the phylogenetic relationships were built with random fragment combinations, optimal evolutionary tree with high supporting values were established using the combinations of *rbcL* + *matK* + *trnH*-*psbA* in tropical cloud forests.

## Introduction

DNA barcoding is a standard gene fragment^1^ for species identification. It has been developing rapidly in recent years^2^, and become a useful tool for biodiversity investigation and monitoring, and molecular phylogeny and evolution^3^.

In 2009, the Consortium for the Barcode of Life (CBOL) Plant Working Group proposed the chloroplast gene *rbcL* and *matK* as the core barcodes of plant species, as well as intergenic sequence *trnH*-*psbA* and nuclear gene ITS as the supplement barcodes^4^. Since *rbcL* is characterized by its universality, easy amplification and comparability, this gene has been proposed as the barcode fragment^5^. Presently, *rbcL* genes have been widely used for phylogenetic analysis within family and subclass of angiosperm, and even among the different groups of the seed plants^6^. However, variation in *rbcL* sequence mainly exists at the above-species level, and variation is seldom found at the species level^7–10^, resulting in poor abilities in species discrimination^4,11^. For example, Newmaster *et al*. compared ~10,300 *rbcL* sequences (with each more than 1,000 bp) collected from GenBank by using a distance method, and found that *rbcL* did not recognize all plant species but distinguished plants within the same genus^10^.

The core barcode *matK* locates at the intron region in chloroplast lysine tRNA (*trnK*) gene, and is ~1,550 bp in length, encoding a mature enzyme that involves in type II intron splicing during RNA transcripts^12^. *matK* is a single-copy and one of the fastest evolving g“enes in protein encoding regions of the chloroplast genome^12^. The evolution rate of this gene is about 2–3 times higher than *rbcL* ^13^, and half lower than the ITS sequence^14^. Although the amplification success rate of *matK* is relative low^7,8,10^, it has been commonly used in studies of systematic and evolutionary botany^15,16^. For example, Lahaye *et al*. studied 1,667 plant samples by using *matK*, and obtained an amplification rate of 100%, and a species discrimination rate of over 90%^17^.

*trnH*-*psbA* sequence locates at intergenic (non-coding) region in chloroplast with a rapid evolution rate. There are 75-bp conserved regions at the two ends of this sequence and makes it easy to be designed as universal primers^18^. *TrnH*-*psbA* sequence has been successfully amplified in many plant species, and showed a high power on discrimination^10^. For example, Kress *et al*. found that the length of the amplified *trnH*-*psbA* fragments of 92% species ranged from 340 to 660 bp, and retained a unique interval sequence, making this sequence meet the criteria as a barcode^19^. However, insertion/deletion events often occur in this fragment in different species^17,19^, even in species that are genetically related^20^, leading to variation in fragment length, and causing difficulties in comparing species from different genera.

ITS belongs to ribosomal DNA in the nuclear genome, and is widely distributed in photosynthetic eukaryotic organisms (except ferns). A large amount of data of this fragment has been accumulated in GenBank^5^, and has become the most common sequence for phylogeny construction^19^. Components of ITS include ITS1, ITS2 and 5.8 S. Experimental evidence shows that there are large differences among the three sequences. For example, 5.8 S is the most conserved gene among the three sequences, and the discrimination power of ITS1 is higher than ITS2^21^. The wide applications of ITS result from the following advantages: (1) ITS is highly repetitive in the nuclear genome, and has high rate of species identification^7^; (2) ITS can be used to solve the problem of plant phylogeny in lower taxonomic order^22^, helping precisely reconstruct phylogenetic relationships between plant species. Li *et al*. successfully got a high species discrimination resolution for ITS by studying the 6,286 samples from 1,757 seed plant species in China^23^; (3) ITS1 and ITS2 locates between 18S and 5.8S rDNA, and 5.8S and 26S DNA respectively. Sequences of 18S, 5.8S and 26S rDNA are highly conserved from bacteria, fungi and higher plants, enabling the design of the sequence-complemented universal primers for PCR amplification of ITS^24^. The use of ITS, however, was also questioned. For example, its success rate of amplification and sequencing was found relatively low (i.e. 86.20% and 71.00% respectively^25^). This usually results from some second-level structures in ITS^26,27^, which makes the sequencing quality of ITS decrease. Secondly, variation in the length of ITS is large, with most sequences longer than 1,100 bp and preserving long sequence of poly-G, poly-C and poly-A, often bringing some difficulties in sequence analysis^8^.

The APG system is always chosen to establish phylogenetic relationships among plant species. But the method cannot distinguish evolutionary relatedness at the species level, and thus processes low resolution of evolutionary trees^28,29^. DNA barcoding provides a new insight into solving this task. With this strategy, desired evolutionary relationships of different plant species within the same community can be constructed when standard DNA fragments are prepared^30^. For example, the optimal evolutionary relationships of tree species in Barro Colorado Island (BCI)^31^ and the Ailao Mountain^32^ have been constructed by integrating fragments *rbcL*, *matK* and *trnH*-*psbA*. Another study, however, suggested that *rbcL* + ITS2 fragment combination can be used as an effective way testing the phylogenetic relationships in Dinghu mountain^33^; and a combination of *trnH*-*psbA* + ITS fragment is favorable for identifying species in Xishuangbanna National Nature Reserve^25^. These cases suggest that DNA barcode combinations can be variable when building phylogenetic relationships of species deriving from different forest communities.

Tropical cloud forest is frequently covered by cloud in humid tropics areas^34^, which mainly distributed at the ridge of mountains with altitudes of 500–3900 m in tropical areas of America, Africa and Asia. Environmental conditions in these forests are characterized by strong wind, low temperature, frequent fog, and high levels of ultraviolet radiation compared with lower altitude forests^35^. Trees in cloud forests are typically more malformed and elfin, and covered in more epiphytes^36^. Endemic and threatened species are rich in tropical cloud forests^37^, and their function in capturing water condensed from clouds and fog^38^, all contribute to the unique ecology of such cloud forest ecosystems^39^. Therefore, the phylogenetic relationships of tree species in this forest community were assumed to differ from low-altitudinal tropical forests, and could not be precisely constructed with the existed APG system. In this paper, we tested the phylogenetic relationships of tree species in tropical cloud forests in Hainan Island through the analysis of DNA barcodes including ITS, *matK*, *rbcL* and *trnH*-*psbA*. We aimed that (1) ITS, *matK*, *rbcL* and *trnH*-*psbA* would be universally used as DNA barcodes for tree species in tropical cloud forests in Hainan Island, and would identify tree species; (2) phylogenetic relationships would be successfully built by using the combination of the four fragments in the tropical cloud forests.

## Results

### Universality of primer sequences

In the tropical cloud forest of Bawangling, samples of a total of 186 individuals and 107 tree species were collected, and 548 sequences were available for the four DNA fragments (Table 1). Among these fragments, *trnH-psbA* had the highest success rate of PCR amplification (83.87%), followed by *rbcL* (80.37%) and *matK* (59.63%), and the success rate of PCR amplification for ITS was the lowest (58.23%). Regarding DNA sequencing, *rbcL* and *trnH-psbA* showed the highest success rate (82.80% and 68.28%, respectively), followed by ITS (56.99%) and *matK* (50%).

**Table 1:** The success rates of PCR amplification and sequencing of the four barcode fragments in the three tropical cloud forests.

Plot	DNA fragment	PCR amplification success rate (%)	Sequencing success rate (%)
Bawangling	ITS	58.23	56.99
	*rbcL*	80.37	68.28
	*matK*	59.63	50.00
	*trnH-psbA*	83.87	82.80
Limushan	ITS	40.35	39.56
	*rbcL*	69.78	57.36
	*matK*	61.47	57.36
	*trnH-psbA*	68.66	55.81
Jianfengling	ITS	52.36	46.67
	*rbcL*	75.62	65.88
	*matK*	50.61	45.10
	*trnH-psbA*	85.32	80.00

In the tropical cloud forest of Limushan, samples of 130 individuals and 89 species were collected, and 356 sequences from the four DNA fragments were available. The success rate of PCR amplification was the highest for *rbcL* (69.78%), followed by *trnH*-*psbA* (68.66%) and *matK* (61.47%). 40.35% of samples was successfully amplified for ITS. *rbcL* and *matK* had the highest success rate of sequencing (57.36%), followed by *trnH*-*psbA* (55.81%) and ITS (39.56%).

In the tropical cloud forest of Jianfengling, samples of 255 individuals belonging to 128 species were collected, and 776 sequences of the four DNA fragments were obtained. A highest success rate of amplification was recorded for *trnH-psbA* (85.32%), followed by *rbcL* (75.62%), ITS (52.36%) and *matK* (50.61%). A highest rate of samples was successfully sequenced for *trnH-psbA* (80.00%), followed by *rbcL* (65.88%), ITS (46.67%), and *matK* (45.10%).

### Success rate of species identification

**Figure 1:**
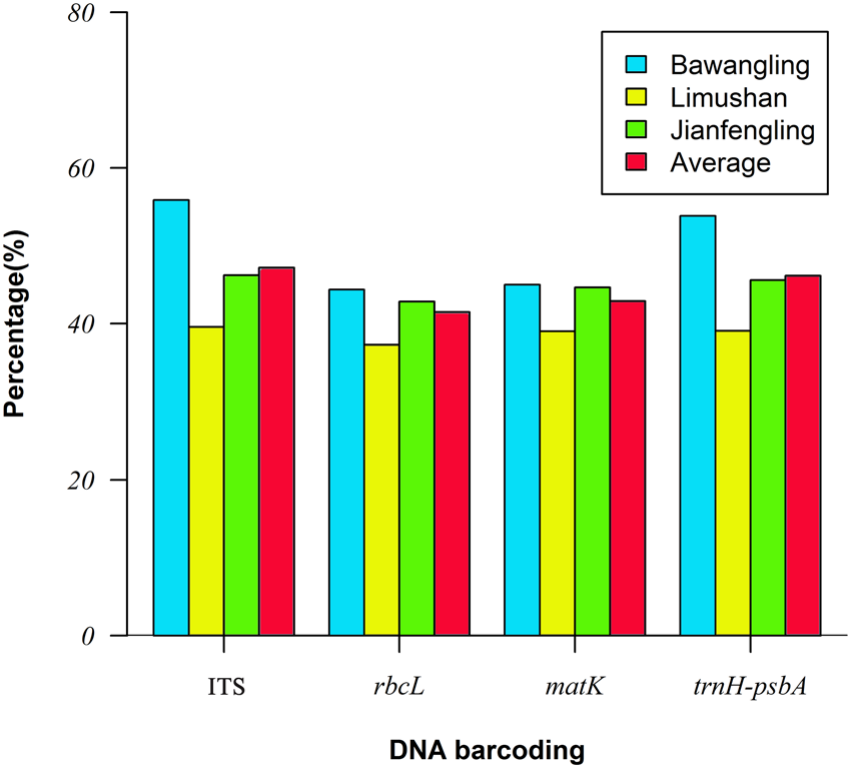
The average species identification success rate for the four barcode fragments in tropical cloud forests.

When single DNA fragment was used, the highest success rate of species identification of 47.20 ± 5.76% was obtained for ITS, followed by *trnH*-*psbA* (46.16 ± 5.11%) and *matK* (42.88% ± 2.59%). But the rate of *rbcL* was the lowest (41.50% ± 2.81%) (Fig. 1). Plants belonging to Lauraceae, Fagaceae, Aquifoliaceae and Symplocaceae, however, could not be effectively identified using a single fragment.

### Construction of phylogenetic trees

The phylogenetic relationships in the tropical cloud forests of Bawangling, Jianfengling, and Limushan were established by the combination of *rbcL* + *matK* + *trnH*-*psbA* (Figs 2–4), with the high average supporting values for nodes (e.g. Bawangling: 79.10% ± 17.87%; Limushan: 76.82% ± 15.69%; Jianfengling: 78.98% ± 14.50%). But the average supporting values for nodes of evolutionary trees in the three forests were relative low when using the other fragment combinations (Suplementary Figs S1–S30, Table S4). Each tree showed a “fan” shape, with closely related species clustering together, whereas distantly related species relatively scattering. Compared with the Limushan and Jianfengling, higher average supporting values were found for nodes on the phylogeny in Bawangling.

**Figure 2:**
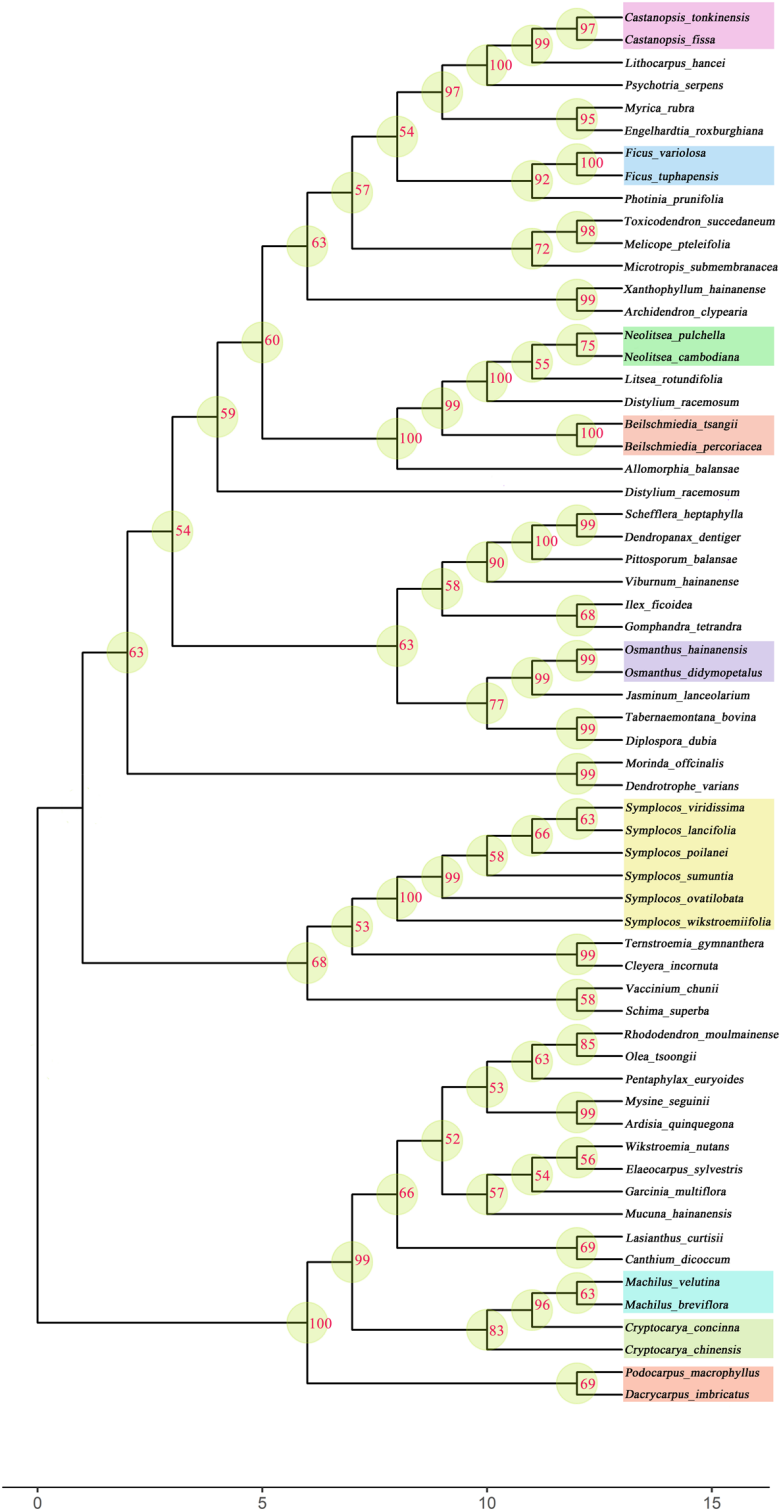
The phylogenetic tree of Bawangling tropical cloud forest using fragment combination of *rbcl* + *matk* + *trnH-psbA*.

**Figure 3:**
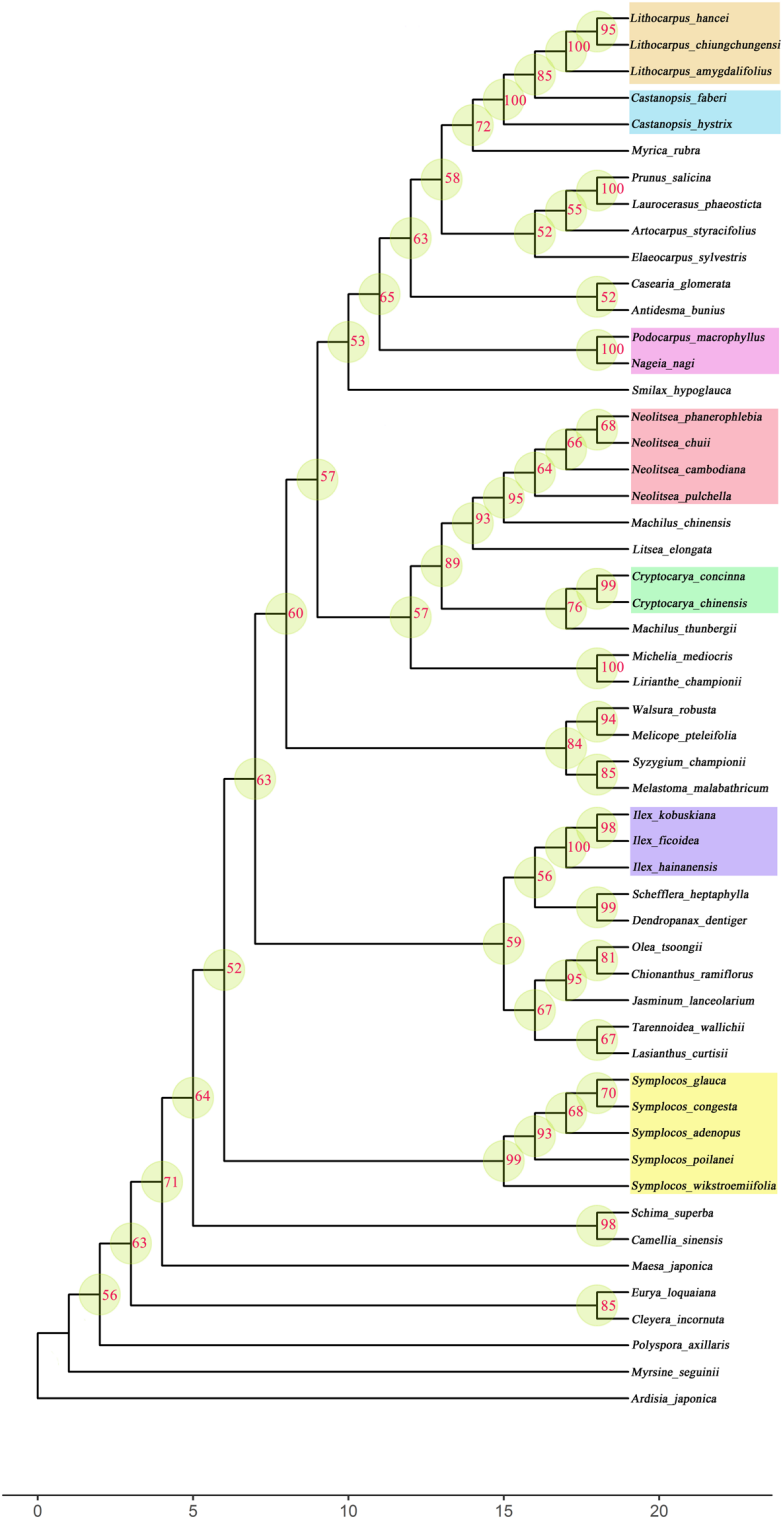
The phylogenetic tree of Limushan tropical cloud forest using fragment combination of *rbcl* + *matk* + *trnH-psbA*.

**Figure 4:**
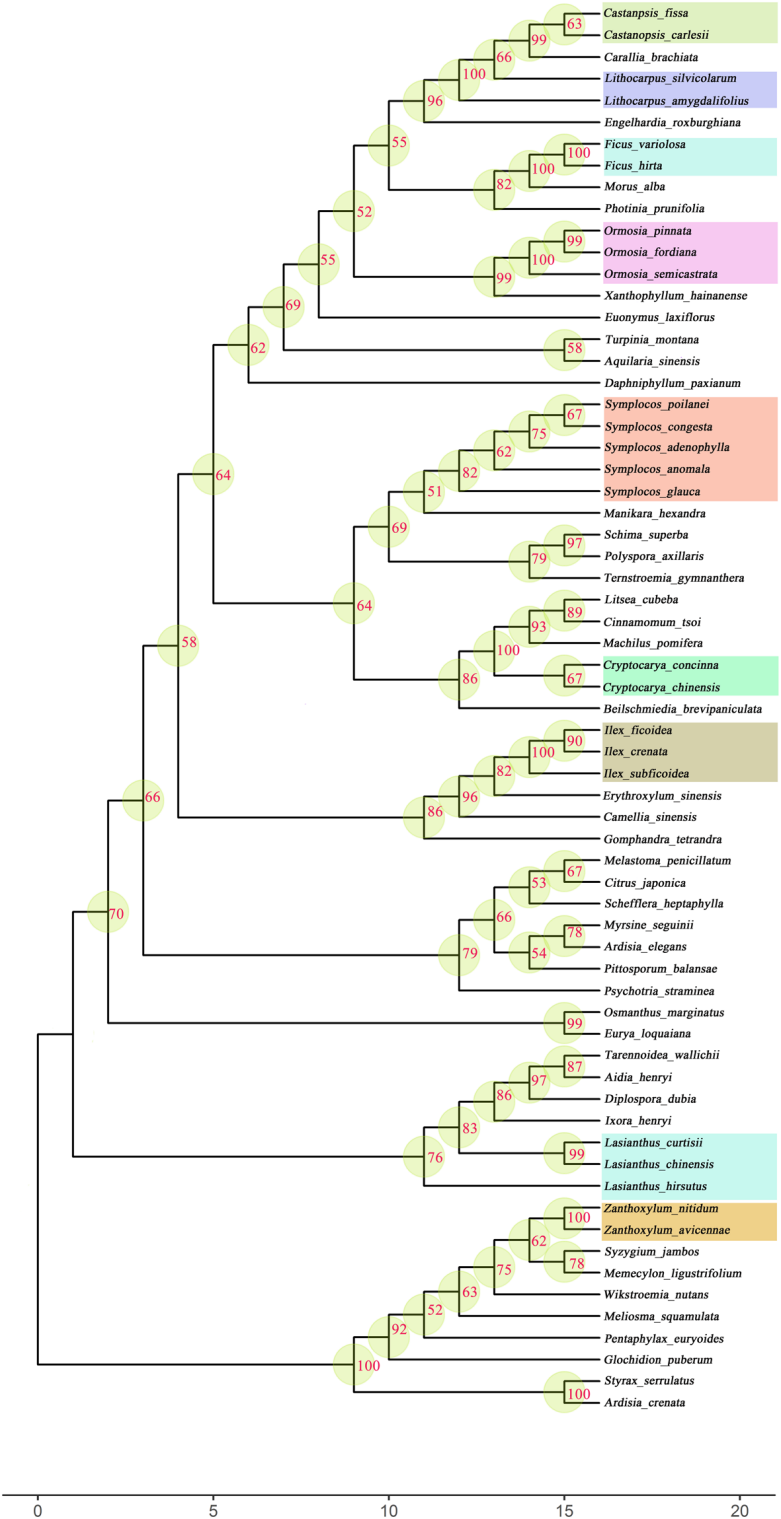
The phylogenetic tree of Jianfengling tropical cloud forest using fragment combination of *rbcl* + *matk* + *trnH-psbA*.

## Discussion

In the present study, the success rates of amplification and sequencing of *matK* fragment were 57.24 ± 4.42% and 50.82 ± 4.36%, respectively, similar to by Kress *et al*.^31^, who reported the *matK* had the lowest overall rate of recovery (69%). The success rates of amplification in Kress *et al*.^7^ was only 39.3% for the 96 species in 46 genera, and the correct recognition rate was 14.6%. Sass *et al*.^8^ used *matK* to amplify *Cycas*, with a success rate of only 24%. Different branch groups of the gene are hard to amplificate and sequencing primers universality is very poor^6,40^. The universality of primers is recognized as an important criteria for evaluating the appropriateness of DNA barcodes^4,41^. The low success rate of amplification and sequencing of *matK* fragments probably shows that it has a poor universality. This is possibly caused by in-sufficient number of primer pairs selected, and can be solved by using more and diverse primers. For example, Lu *et al*. included additional primers *matK472F* and *matK1248R* in the DNA barcode candidates^32^, and obtained a 90% of the universality of primers for subtropical forest tree species in Ailao mountain.

High success rate of amplification and sequencing (75.26% ± 3.65% and 63.84% ± 4.32%, respectively) was found for *rbcL* in the tropical cloud forest, which is consistent with the study in Xishuangbanna Nature Reserve^25^ (75.26% ± 3.65% vs. 97.60% and 63.84% ± 4.32%vs. 90.80%, respectively), indicating a promise universality for the *rbcL* barcode. The result is thought to be related to highly conserved and low evolutionary levels for *rbcL* gene. Although high success rate of amplification (79.28% ± 7.08%) can also be obtained for *trnH*-*psbA*, sequence data were generally enriched by repeated sequences. This may result from the evidence that there are mononucleotide repeats in *trnH*-*psbA* gene in some species, with continuous repeats of several to dozen bases of A or T. Moreover, the uncertainty number of nucleotide repeat sequences in *trnH*-*psbA* was prone to be caused by traditional Sanger sequencing method^42^.

In this study, ITS showed the lowest rate of amplification and sequencing (50.31% ± 6.64% and 45.15% ± 8.91%, respectively) in all of the fragments tested. The success rate of amplification and sequencing of ITS can be variable for tropical forest plants in different regions. For instance, only 41% of success rate of ITS was detected in 285 tree species in the Amazon tropical forests^11^. For the tree species from India tropical forests, however, the rates of amplification and sequencing for ITS were 74% and 62%, respectively^43^. The low rate of amplification and sequencing of tropical cloud forest species can be partly explained by Lauraceae and Fagaceae species, which contain large amount of secondary metabolites (such as polysaccharides and phenolic compounds) that has a negative effect on the extraction of high quality DNA. Moreover, multiple copies of fragments in ITS, as well as its incomplete concerted evolution process in *Quercus* species^44,45^ and *Cinnamomum* species^46^, may also be responsible for the rate of amplification and sequencing.

The identification success rates of *rbcL* and *matK* were 41.50% ± 2.81% and 42.88% ± 2.59% in tropical cloud forests, respectively, lower than those of *trnH-psbA* and ITS (Fig. 1). This may result from the fact that variation in *rbcL* sequence mainly exists at the above-species level^7–10^, as well as the relative low evolution rate of Johnson & Soltis^14^. Low identification success rate for both *rbcL* and *matK* have also be found by Huang *et al*. in the tropical rain forest in Xishuangbanna in China^25^, and by Tarnowski *et al*. in the tropical rain forest in India^43^. Therefore, our results suggest that both rbcL and matK be not suitable for identification of plants at the species level in tropical forests. But *rbcL* and *matK* have the advantage of high success rate of amplification and sequencing in tropical cloud forests (Fig. 1), as well as advantage of that these two fragments have been demonstrated to have high identification success rates at the genus and family level^25^, we thus propose that both *rbcL* and *matK* be two core barcodes identifying plant evolutionary relationships at the genus and family levels in tropical cloud forests.

Although there is a lot of debate about whether *trnH-psbA* fragments can be used as DNA barcodes^1,47–49^, we found that the identification rate of this fragment was 46.16% ± 5.11%. Our results are consistent with the finding of Huang *et al*. in the tropical rain forest in Xishuangbanna^25^, and show that *trnH*-*psbA* is a good candidate used as species identification in tropical cloud forest. Especially, our study shows that *trnH*-*psbA* can be used as a barcode for tree species belonging to Lauraceae and Fagaceae, when these species are seldom identified by the other three fragments. Other studies have shown that *trnH-psbA* can be used to identify plant species individually^50^, or is taken as auxiliary barcodes^51^. Thus our findings prove that *trnH*-*psbA* is a potential DNA barcode for forest tree species identification^4,11,43^.

The identification rate of ITS in the tropical cloud forest was 47.20 ± 5.76%, lower than the data of Li *et al*.^23^ (BLAST: ITS 67.20%) and Huang *et al*.^25^ (BLAST: ITS 58.10%). The relative low identification rate of ITS in tropical cloud forests may result from the low rate of amplification and sequencing of this fragment. But the highest species discrimination rate of this fragment in the tropical cloud forests probably shows that it is a core barcode of angiosperm plant. Other evidence demonstrated that ITS acted as one of the standard DNA barcodes identifying global land plants^6^ and green algae^52^. Thus, our results further prove that ITS is a plant core barcode which can effectively identify plant species.

We successfully constructed phylogenetic relationships of tree species using *rbcL* + *matK* + *trnH*-*psbA* fragment combinations in these three tropical cloud forests, which is contrary to our second hypothesis. Moreover, we found that the average supporting values of nodes on each branch were higher than 50% (Figs 2–4), indicating that we obtain highly reliable evolutionary relationships for tropical cloud forest tree species. The *rbcL* + *matK* + *trnH*-*psbA* fragments were previously used to construct phylogenetic relationships for tree species in Barro Colorado Island (BCI), Dinghu Mountains and Ailao Mountain forest^31,32,53^. And studies have shown that the combination of *rbc*L + *mat*K + *trn*H-*psb*A is currently applicable for DNA barcoding-based phylogenetic studies on forest communities^54^. Our results further prove that these three fragments are of high efficiency in reconstructing phylogenetic relationships for forest plant species. Since tropical cloud forest has rich species and endemic species, the phylogenetic tree constructed by the APG online system (constrained tree) is difficult to reflect the phylogenetic relationships in the tropical cloud forests. Phylogenetic trees constructed by DNA sequences (non-constrained tree) in this study are able to clearly cluster the closely related species and separate distantly related species in tropical cloud forest (Figs 2–4).

We found a large number of Symplocaceae, Fagaceae and Lauraceae species in the three tropical cloud forests, indicating a similar species composition. These results may reflect similar origin and phylogenetic framework of tropical cloud forest species in Hainan Island. However, the three tropical cloud forests have different environmental conditions due to their different geographical locations. For example, Jianfengling (southwest Hainan Island) has the highest mean temperature, followed by Bawangling (Western Hainan Island), and Limu mountain (central Hainan Island) is the lowest. These differences may be responsible for the different direction of species evolution in the three tropical cloud forests. For example, *Distylium racemosum* is dominant in Bawangling^35^, and *Camellia pitardii* is dominant in Limushan, whereas Fagaceae plants such as *Castanopsis faberi* are more widely distributed in Jianfengling^55^.

In general, contrasting with the first hypothesis, we found that *matK* and ITS had low success rates of amplification and sequencing (Table 1), showing that these two fragments have poor universality in the tropical cloud forests. But we found high success rate of amplification and sequencing for *rbcL* and *trnH-psbA* (Table 1), indicating that these two barcodes are universal for tree species in the tropical cloud forests. Similar to other studies, both *rbcL* and *matK* had low rates of species discrimination rate (Fig. 1), but high identification success rates at the genus and family level, suggesting that these two fragments are core barcodes identifying genus- and family-level evolutionary relationships for tropical cloud forest plants. The species identification rates of *trnH-psbA* and ITS were relative high among the four fragments (Fig. 1), showing that they are good candidates used as species identification in tropical cloud forest. Contrary to the second hypothesis, we constructed highly reliable evolutionary relationships for tropical cloud forest tree species using a three- fragment combination (Figs 2–4; *rbcL* + *matK* + *trnH-psbA*), similar to the results in Barro Colorado Island (BCI), Dinghu Mountains and Ailao Mountain forest^31,32,53^. Our results thus prove that the three fragment combinations are of high efficiency in reconstructing phylogenetic relationships for forest plant species.

## Materials and Methods

### Sample collection

In 2013 and 2014, we established 12, 21 and 15 20 × 20-m plots in tropical cloud forests in Jianfengling, Bawangling and Limushan, respectively (Table 2). Fresh and intact leaves were collected from 1–2 individual trees for each species with diameter at breast height (DBH) more than 5 cm, in the wake of drying treatment by using silica gel^23^.

**Table 2:** Description of the study sites.

Plot	Number	Area (m^2^)	Species Number	Elevation (m)
Bawangling	21	8400	107	1340
Limushan	15	6000	89	1411
Jianfengling	12	4800	128	1280

### DNA extraction, amplification and sequencing

Genomic DNA was extracted by using a Plant DNA Isolation Kit (Foregene, Chengdu, China), and four fragments including ITS, *rbcL*, *matK*, and *trnH*-*psbA* were selected (Suplementary Table S1). PCR reaction system was optimized and modified based on the recommended protocol^4^. Amplification products were sent to Huada Genomics Institute (BGI, Guangzhou, China) for DNA sequencing. Sequence editing and alignment, barcode assembly, and the construction of evolutionary trees were preformed by using BioEdit programs (http://www.softpedia.com/get/Science-CAD/BioEdit.shtm), Sequencematrix (http://www.softpedia.com/get/Science-CAD/Sequence-Matrix.shtml), and MEGA 6.0 (http://www.megasoftware.net/).

### Data analysis

The success rate of PCR amplification was calculated as the proportion of the number of individuals amplified to the total number of individuals analyzed, and the successful rate of sequencing was calculated as the percentage of the number of high quality sequences to the total number of individuals^31^.

Species identification ability of each DNA barcodes was evaluated using the BLAST method. The work was conducted as follows: firstly, each DNA fragment of the collected species in tropical cloud forests was downloaded from NCBI database, and then a local database was established using the downloaded sequences^56^. Secondly, each sequence measured in this study was BLAST against the sequence in the local database, and the percentage of identical sites was calculated and was taken as the species discrimination rate of the measured sequence. If the percentage of identical sites of a sequence calculated between intraspecific individuals were higher than interspecific individuals, then the sequence was taken as the purpose one of the studied species. Finally the identification success rate of DNA barcoding was calculated as the product of sequencing success rate and species discrimination rate^31^.

Methods for constructing evolutionary trees mainly include discrete character methods and distance methods. Discrete character methods include minimum evolution (ME) method and maximum likelihood (ML) method, while distance methods include the unweighted pair-group method with arithmetic means (UPGMA) and neighbor joining (NJ) method. The two methods of ME and ML have long computation time and complex computation. And the UPGMA algorithm is simpler and is rarely used at present. However, the NJ method can deal with a large amount of sequence information on a personal computer, and a bootstrap test can be easily performed. This method was used to construct phylogenetic trees in the present study, because trees constructed by NJ method meet the requirements for species identification. Lahaye *et al*.^17^ demonstrated that the optimal topology was easily generated when evolutionary tree was built by using NJ method.

## Electronic supplementary material

Supporting Information
