# Supplementary material for: DNA barcoding analysis and phylogenetic relationships of tree species in tropical cloud forests
Source: Sci Rep. 2017 Oct 2;7:12564. doi: 10.1038/s41598-017-13057-0 (PMC5624878; doi:10.1038/s41598-017-13057-0)
Supplement: Supplementary file 1 — Supporting Information [file 41598_2017_13057_MOESM1_ESM.doc]

**Supporting Information**

**DNA barcoding analysis and phylogenetic relationships of tree species in tropical cloud forests**

Yong Kang ^a^, Zhiyan Deng ^a^, Runguo Zang^b^, Wenxing Long^*a^

^a^ Hainan Key Laboratory for Sustainable Utilization of Tropical Bioresource; Institute of Tropical Agriculture and Forestry, Hainan University, Haikou, 570228, China.

^b^ Key Laboratory of Forest Ecology and Environment of State Forestry Administration; Institute of Forest Ecology, Environment and Protection, Chinese Academy of Forestry, Beijing, 100091, China.

* Correspondence author. Email: Wenxing Long, [oklong@hainu.edu.cn](mailto:oklong@hainu.edu.cn)

**Table S1. Information of the four DNA fragments**

| DNA fragment | Primer pair | Sequence (5ˊ– 3ˊ) | Reference |
| --- | --- | --- | --- |
| ITS | ITS4 | TCCTCCGCTTATTGATATGC | White et al. |
|  | ITS5 | GGAAGTAAAAGTCGTAACAAGG | White et al. |
| *rbcL* | *rbcL724R* | TCGCATGTACCTGCAGTAGC | Ivanova et al. |
|  | *rbcL1F* | ATGTCACCACAAACAGAGACTAAAGC | Ivanova et al. |
| *matK* | *matK3F* | CGTACAGTACTTTTGTGTTTACGAG | Kim ( unpublished) |
|  | *matK1R* | ACCCAGTCCATCTGGAAATCTTGGTTC | Kim ( unpublished) |
| *trnH-psbA* | *trnH* | CGCGCATGGTGGATTCACAATCC | Fazekas et al. |
|  | *psbA* | GTTATGCATGAACGTAATGCTC | Sang et al. |

**Table S2. The species list of Bawangling** Mountain

| **Species** | **Genus** | **Family** |
| --- | --- | --- |
| *Schefflera heptaphylla* | *Schefflera* | Araliaceae |
| *Canthium dicoccum* | *Canthium* | Rubiaceae |
| *Podocarpus macrophyllus* | *Podocarpus* | Podocarpaceae |
| *Diplospora dubia* | *Diplospora* | Rubiaceae |
| *Castanopsis tonkinensis* | *Castanopsis* | Fagaceae |
| *Gomphandra tetrandra* | *Gomphandra* | Icacinaceae |
| *Lasianthus curtisii* | *Lasianthus* | Rubiaceae |
| *Beilschmiedia percoriacea* | *Beilschmiedia* | Lauraceae |
| *Distylium racemosum* | *Distylium* | Hamamelidaceae |
| *Ternstroemia gymnanthera* | *Ternstroemia* | Theaceae |
| *Ficus variolosa* | *Ficus* | Moraceae |
| *Myrsine seguinii* | *Myrsine* | Myrsinaceae |
| *Symplocos wikstroemiifolia* | *Symplocos* | Symplocaceae |
| *Photinia prunifolia* | *Photinia* | Rosaceae |
| *Myrica rubra* | *Myrica* | Myricaceae |
| *Ilex hainanensis* | *Ilex* | Aquifoliaceae |
| *Olea tsoongii* | *Olea* | Oleaceae |
| *Litsea rotundifolia* | *Litsea* | Lauraceae |
| *Wikstroemia nutans* | *Wikstroemia* | Thymelaeaceae |
| *Mucuna hainanensis* | *Mucuna* | Fabaceae |
| *Archidendron clypearia* | *Archidendron* | Fabaceae |
| *Microtropis submembranacea* | *Microtropis* | Celastraceae |
| *Xanthophyllum hainanense* | *Xanthophyllum* | Polygalaceae |
| *Elaeocarpus sylvestris* | *Elaeocarpus* | Elaeocarpaceae |
| *Dendrotrophe varians* | *Dendrotrophe* | Santalaceae |
| *Beilschmiedia tsangii* | *Beilschmiedia* | Lauraceae |
| *Pittosporum balansae* | *Pittosporum* | Pittosporaceae |
| *Neolitsea cambodiana* | *Neolitsea* | Lauraceae |
| *Cleyera incornuta* | *Cleyera* | Theaceae |
| *Ilex ficoidea* | *Ilex* | Aquifoliaceae |
| *Neolitsea pulchella* | *Neolitsea* | Lauraceae |
| *Dendropanax dentiger* | *Dendropanax* | Araliaceae |
| *Allomorphia balansae* | *Allomorphia* | Melastomataceae |
| *Ardisia quinquegona* | *Ardisia* | Myrsinaceae |
| *Castanopsis fissa* | *Castanopsis* | Fagaceae |
| *Cryptocarya chinensis* | *Cryptocarya* | Lauraceae |
| *Cryptocarya concinna* | *Cryptocarya* | Lauraceae |
| *Morinda officinalis* | *Morinda* | Rubiaceae |
| *Engelhardtia roxburghiana* | *Engelhardtia* | Juglandaceae |
| *Pentaphylax euryoides* | *Pentaphylax* | Pentaphylacaceae |
| *Melicope pteleifolia* | *Melicope* | Rutaceae |
| *Osmanthus didymopetalus* | *Osmanthus* | Oleaceae |
| *Dacrycarpus imbricatus* | *Dacrycarpus* | Podocarpaceae |
| *Machilus velutina* | *Machilus* | Lauraceae |
| *Symplocos lancifolia* | *Symplocos* | Symplocaceae |
| *Jasminum lanceolarium* | *Jasminum* | Oleaceae |
| *Lithocarpus hancei* | *Lithocarpus* | Fagaceae |
| *Symplocos poilanei* | *Symplocos* | Symplocaceae |
| *Rhododendron moulmainense* | *Rhododendron* | Ericaceae |
| *Machilus breviflora* | *Machilus* | Lauraceae |
| *Toxicodendron succedaneum* | *Toxicodendron* | Anacardiaceae |
| *Viburnum hainanense* | *Viburnum* | Adoxaceae |
| *Psychotria serpens* | *Psychotria* | Rubiaceae |
| *Heterosmilax japonica* | *Heterosmilax* | Liliaceae |
| *Schima superba* | *Schima* | Theaceae |
| *Ficus tuphapensis* | *Ficus* | Moraceae |
| *Vaccinium chunii* | *Vaccinium* | Ericaceae |
| *Symplocos viridissima* | *Symplocos* | Symplocaceae |
| *Tabernaemontana bovina* | *Tabernaemontana* | Apocynaceae |
| *Symplocos sumuntia* | *Symplocos* | Symplocaceae |
| *Microtropis submembranacea* | *Microtropis* | Celastraceae |
| *Garcinia multiflora* | *Garcinia* | Clusiaceae |
| *Osmanthus hainanensis* | *Osmanthus* | Oleaceae |
| *Symplocos ovatilobata* | *Symplocos* | Symplocaceae |

**Table S3. The species list of Limushan** Mountain

| **Species** | **Genus** | **Family** |
| --- | --- | --- |
| *Ardisia japonica* | *Ardisia* | Myrsinaceae |
| *Neolitsea pulchella* | *Neolitsea* | Lauraceae |
| *Ilex kobuskiana* | *Ilex* | Aquifoliaceae |
| *Polyspora axillaris* | *Polyspora* | Theaceae |
| *Machilus chinensis* | *Machilus* | Lauraceae |
| *Dendropanax dentiger* | *Dendropanax* | Araliaceae |
| *Michelia mediocris* | *Michelia* | Magnoliaceae |
| *Elaeocarpus sylvestris* | *Elaeocarpus* | Elaeocarpaceae |
| *Lithocarpus chiungchungensis* | *Lithocarpus* | Fagaceae |
| *Melastoma malabathricum* | *Melastoma* | Melastomataceae |
| *Lithocarpus amygdalifolius* | *Lithocarpus* | Fagaceae |
| *Casearia glomerata* | *Casearia* | Flacourtiaceae |
| *Cryptocarya chinensis* | *Cryptocarya* | Lauraceae |
| *Olea tsoongii* | *Olea* | Oleaceae |
| *Lasianthus curtisii* | *Lasianthus* | Rubiaceae |
| *Maesa japonica* | *Maesa* | Myrsinaceae |
| *Cleyera incornuta* | *Cleyera* | Theaceae |
| *Walsura robusta* | *Walsura* | Meliaceae |
| *Castanopsis hystrix* | *Castanopsis* | Fagaceae |
| *Lithocarpus hancei* | *Lithocarpus* | Fagaceae |
| *Myrsine seguinii* | *Myrsine* | Myrsinaceae |
| *Litsea elongata* | *Litsea* | Lauraceae |
| *Symplocos congesta* | *Symplocos* | Symplocaceae |
| *Prunus salicina* | *Prunus* | Rosaceae |
| *Neolitsea phanerophlebia* | *Neolitsea* | Lauraceae |
| *Camellia sinensis* | *Camellia* | Theaceae |
| *Nageia nagi* | *Nageia* | Podocarpaceae |
| *Symplocos wikstroemiifolia* | *Symplocos* | Symplocaceae |
| *Jasminum lanceolarium* | *Jasminum* | Oleaceae |
| *Neolitsea chuii* | *Neolitsea* | Lauraceae |
| *Smilax hypoglauca* | *Smilax* | Liliaceae |
| *Tarennoidea wallichii* | *Tarennoidea* | Rubiaceae |
| *Chionanthus ramiflorus* | *Chionanthus* | Oleaceae |
| *Ilex ficoidea* | *Ilex* | Aquifoliaceae |
| *Machilus thunbergii* | *Machilus* | Lauraceae |
| *Symplocos glauca* | *Symplocos* | Symplocaceae |
| *Neolitsea cambodiana* | *Neolitsea* | Lauraceae |
| *Schefflera heptaphylla* | *Schefflera* | Araliaceae |
| *Castanopsis faberi* | *Castanopsis* | Fagaceae |
| *Ilex hainanensis* | *Ilex* | Aquifoliaceae |
| *Symplocos adenopus* | *Symplocos* | Symplocaceae |
| *Schima superba* | *Schima* | Theaceae |
| *Syzygium championii* | *Syzygium* | Myrtaceae |
| *Cryptocarya concinna* | *Cryptocarya* | Lauraceae |
| *Myrica rubra* | *Myrica* | Myricaceae |
| *Ilex ficoidea* | *Ilex* | Aquifoliaceae |
| *Eurya loquaiana* | *Eurya* | Theaceae |
| *Artocarpus styracifolius* | *Artocarpus* | Moraceae |
| *Laurocerasus phaeosticta* | *Laurocerasus* | Rosaceae |
| *Antidesma bunius* | *Antidesma* | Euphorbiaceae |
| *Podocarpus macrophyllus* | *Podocarpus* | Podocarpaceae |
| *Lirianthe championii* | *Lirianthe* | Magnoliaceae |
| *Symplocos poilanei* | *Symplocos* | Symplocaceae |
| *Melicope pteleifolia* | *Melicope* | Rutaceae |

**Table S4. The species list of Jianfengling** Mountain

| **Species** | **Genus** | **Family** |
| --- | --- | --- |
| *Memecylon ligustrifolium* | *Memecylon* | Melastomataceae |
| *Beilschmiedia brevipaniculata* | *Beilschmiedia* | Lauraceae |
| *Cryptocarya chinensis* | *Cryptocarya* | Lauraceae |
| *Ficus hirta* | *Ficus* | Moraceae |
| *Lasianthus hirsutus* | *Lasianthus* | Rubiaceae |
| *Carallia brachiata* | *Castanopsis* | Fagaceae |
| *Zanthoxylum nitidum* | *Zanthoxylum* | Rutaceae |
| *Lithocarpus silvicolarum* | *Lithocarpus* | Fagaceae |
| *Ormosia semicastrata* | *Ormosia* | Fabaceae |
| *Morus alba* | *Morus* | Moraceae |
| *Symplocos adenophylla* | *Symplocos* | Symplocaceae |
| *Turpinia montana* | *Turpinia* | Staphyleaceae |
| *Ormosia fordiana* | *Ormosia* | Fabaceae |
| *Ormosia pinnata* | *Ormosia* | Fabaceae |
| *Castanopsis fissa* | *Castanopsis* | Fagaceae |
| *Ternstroemia gymnanthera* | *Ternstroemia* | Theaceae |
| *Daphniphyllum paxianum* | *Daphniphyllum* | Daphniphyllaceae |
| *Cryptocarya concinna* | *Cryptocarya* | Lauraceae |
| *Diplospora dubia* | *Diplospora* | Rubiaceae |
| *Ilex ficoidea* | *Ilex* | Aquifoliaceae |
| *Ilex crenata* | *Ilex* | Aquifoliaceae |
| *Erythroxylum sinensis* | *Erythroxylum* | Erythroxylaceae |
| *Camellia sinensis* | *Camellia* | Theaceae |
| *Litsea cubeba* | *Litsea* | Lauraceae |
| *Cinnamomum tsoi* | *Cinnamomum* | Lauraceae |
| *Schefflera heptaphylla* | *Schefflera* | Araliaceae |
| *Ardisia crenata* | *Ardisia* | Myrsinaceae |
| *Citrus japonica* | *Citrus* | Rutaceae |
| *Tarennoidea wallichii* | *Tarennoidea* | Rubiaceae |
| *Symplocos congesta* | *Symplocos* | Symplocaceae |
| *Eurya loquaiana* | *Eurya* | Theaceae |
| *Engelhardia roxburghiana* | *Engelhardia* | Juglandaceae |
| *Pentaphylax euryoides* | *Pentaphylax* | Pentaphylacaceae |
| *Ixora henryi* | *Ixora* | Rubiaceae |
| *Aidia henryi* | *Aidia* | Rubiaceae |
| *Wikstroemia nutans* | *Wikstroemia* | Thymelaeaceae |
| *Styrax serrulatus* | *Styrax* | Styracaceae |
| *Schefflera heptaphylla* | *Schefflera* | Araliaceae |
| *Meliosma squamulata* | *Meliosma* | Sabiaceae |
| *Lasianthus chinensis* | *Lasianthus* | Rubiaceae |
| *Lasianthus curtisii* | *Lasianthus* | Rubiaceae |
| *Ardisia elegans* | *Ardisia* | Myrsinaceae |
| *Symplocos glauca* | *Symplocos* | Symplocaceae |
| *Polyspora axillaris* | *Polyspora* | Theaceae |
| *Ficus variolosa* | *Ficus* | Moraceae |
| *Pittosporum balansae* | *Pittosporum* | Pittosporaceae |
| *Syzygium jambos* | *Syzygium* | Myrtaceae |
| *Ilex subficoidea* | *Ilex* | Aquifoliaceae |
| *Schima superba* | *Schima* | Theaceae |
| *Xanthophyllum hainanense* | *Xanthophyllum* | Polygalaceae |
| *Machilus pomifera* | *Machilus* | Lauraceae |
| *Manilkara hexandra* | *Sapotaceae* | Manilkara |
| *Gomphandra tetrandra* | *Gomphandra* | Icacinaceae |
| *Symplocos anomala* | *Symplocos* | Symplocaceae |
| *Melastoma penicillatum* | *Melastoma* | Melastomataceae |
| *Lithocarpus amygdalifolius* | *Lithocarpus* | Fagaceae |
| *Symplocos poilanei* | *Symplocos* | Symplocaceae |
| *Photinia prunifolia* | *Photinia* | Rosaceae |
| *Zanthoxylum avicennae* | *Zanthoxylum* | Rutaceae |
| *Euonymus laxiflorus* | *Euonymus* | Celastraceae |
| *Psychotria straminea* | *Psychotria* | Rubiaceae |
| *Olea tsoongii* | *Olea* | Oleaceae |
| *Glochidion puberum* | *Glochidion* | Euphorbiaceae |
| *Aquilaria sinensis* | *Aquilaria* | Thymelaeaceae |
| *Castanopsis carlesii* | *Castanopsis* | Fagaceae |
| *Myrsine seguinii* | *Myrsine* | Myrsinaceae |

**Table S5.** **Average** **supporting** **values** **for** **nodes of two, four random fragment** **combinations.**

| **BWL** | Average supporting  values for nodes |
| --- | --- |
| *rbcL+matK+trnH-psbA*+ITS | 72.55%±24.93% |
| *rbcL+matK*+ITS | 70.15%±27.35% |
| *matK+trnH-psbA*+ITS | 71.07%±25.10% |
| *rbcL+ trnH-psbA*+ITS | 28.23%±21.52% |
| *matK* +ITS | 69.03%±27.23% |
| *rbcL* +ITS | 23.40%±18.71% |
| *trnH-psbA*+ITS | 20.50%±18.86% |
| *rbcL+matK* | 72.77%±24.52% |
| *matK+trnH-psbA* | 70.15%±26.36% |
| *rbcL +trnH-psbA* | 29.12%±25.14% |
| **LMS** | Average supporting  values for nodes |
| *rbcL+matK+trnH-psbA*+ITS | 57.48%±29.16% |
| *rbcL+matK*+ITS | 64.44%±26.88% |
| *matK+trnH-psbA*+ITS | 39.10%±29.48% |
| *rbcL+ trnH-psbA*+ITS | 59.00%±30.72% |
| *matK* +ITS | 34.46%±28.13% |
| *rbcL* +ITS | 63.96%±26.92% |
| *trnH-psbA*+ITS | 38.18%±28.66% |
| *rbcL+matK* | 57.90%±30.54% |
| *matK+trnH-psbA* | 21.96%±19.31% |
| *rbcL +trnH-psbA* | 68.26%±24.05% |
| **JFL** | Average supporting  values for nodes |
| *rbcL+matK+trnH-psbA*+ITS | 68.50%±24.60% |
| *rbcL+matK*+ITS | 65.92%±26.06% |
| *matK+trnH-psbA*+ITS | 67.11%±28.01% |
| *rbcL+ trnH-psbA*+ITS | 49.39%±33.32% |
| *matK* +ITS | 68.05%±26.59% |
| *rbcL* +ITS | 45.56%±31.81% |
| *trnH-psbA*+ITS | 26.08%±23.33% |
| *rbcL+matK* | 66.81%±27.48% |
| *matK+trnH-psbA* | 67.10%±28.50% |
| *rbcL +trnH-psbA* | 55.74%±29.20% |


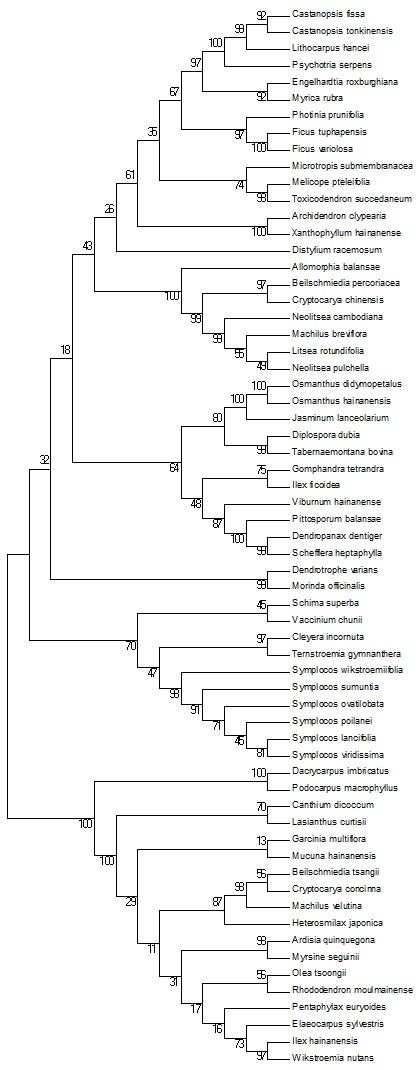


**Fig. S1 The phylogenetic tree of Bawangling tropical cloud forest using fragment combination of *rbcL+matK+trnH-psbA*+ITS**


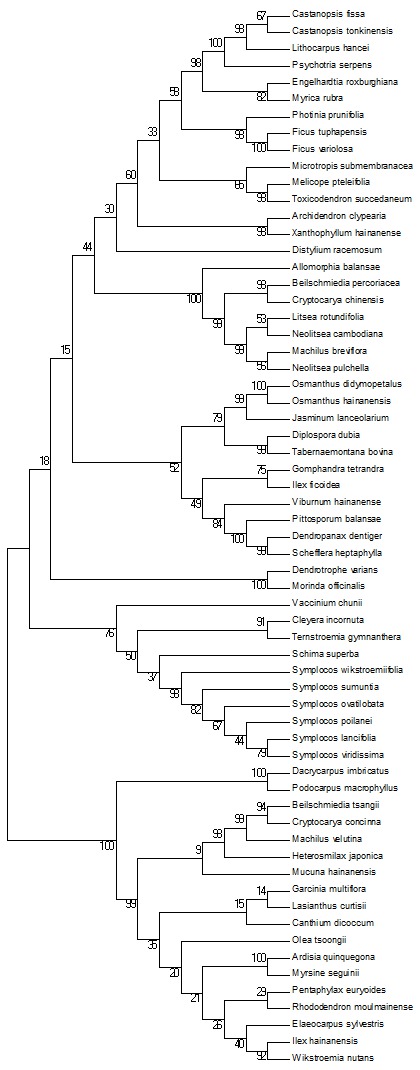


**Fig. S2 The phylogenetic tree of Bawangling tropical cloud forest using fragment combination of *rbcL+matK*+ITS**


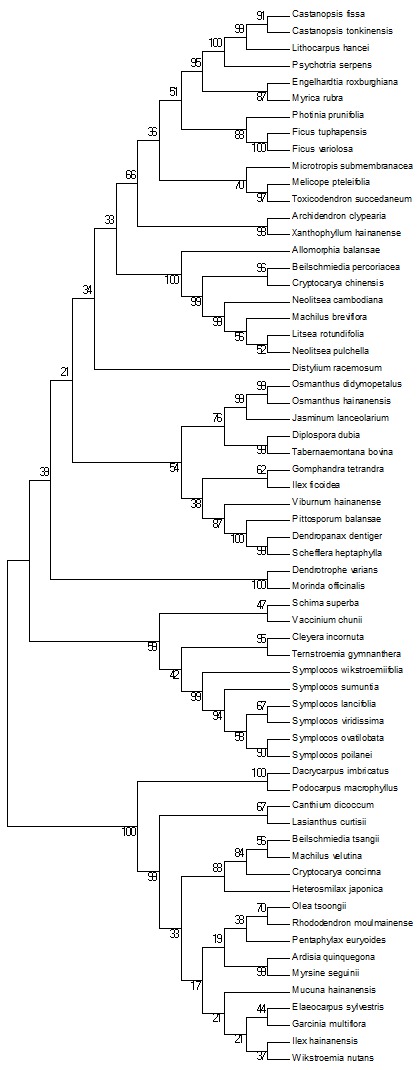


**Fig. S3 The phylogenetic tree of Bawangling tropical cloud forest using fragment combination of *matK+trnH-psbA*+ITS**


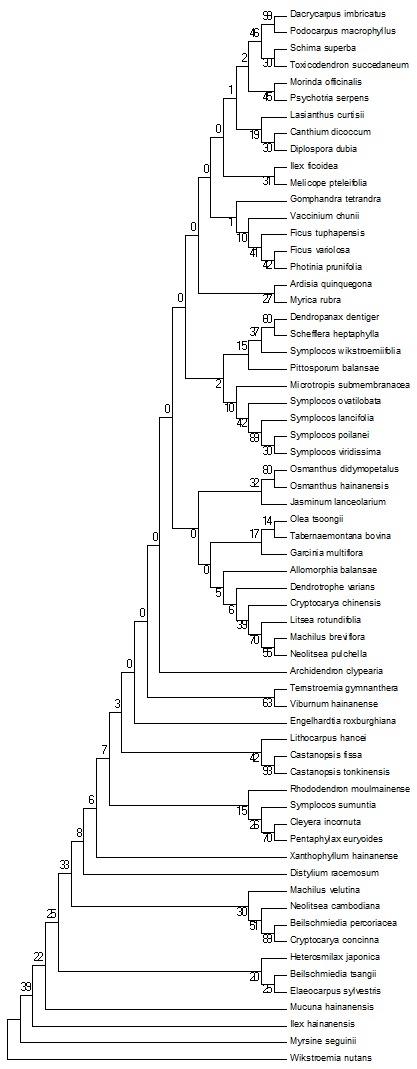


**Fig. S4 The phylogenetic tree of Bawangling tropical cloud forest using fragment combination of *rbcL+ trnH-psbA+*ITS**


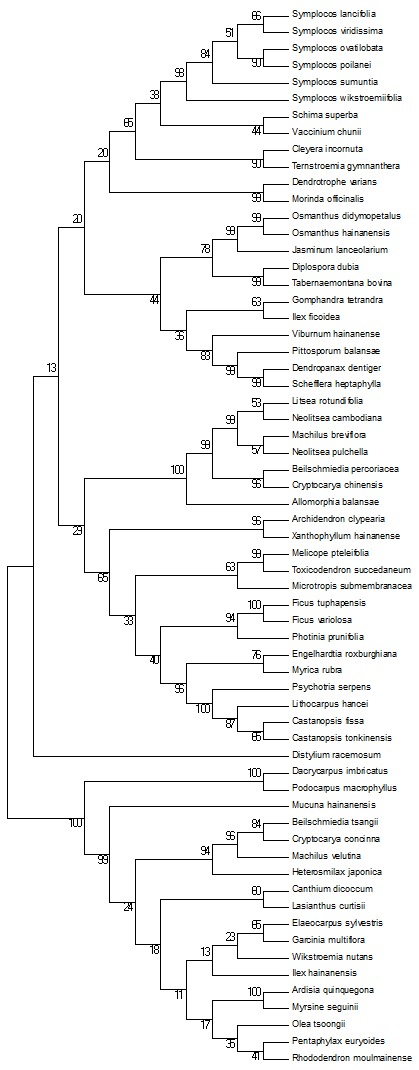


**Fig. S5 The phylogenetic tree of Bawangling tropical cloud forest using fragment combination of *matK* +ITS**


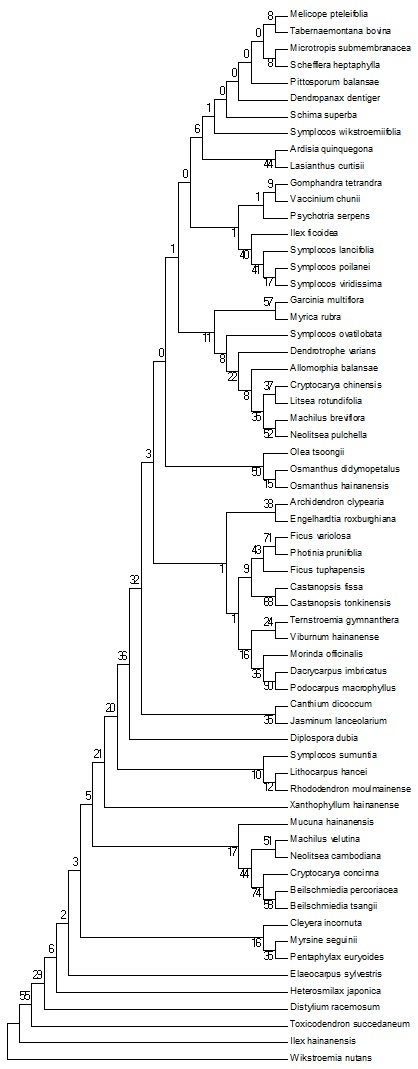


**Fig. S6 The phylogenetic tree of Bawangling tropical cloud forest using fragement combination of *rbcL*+ITS**


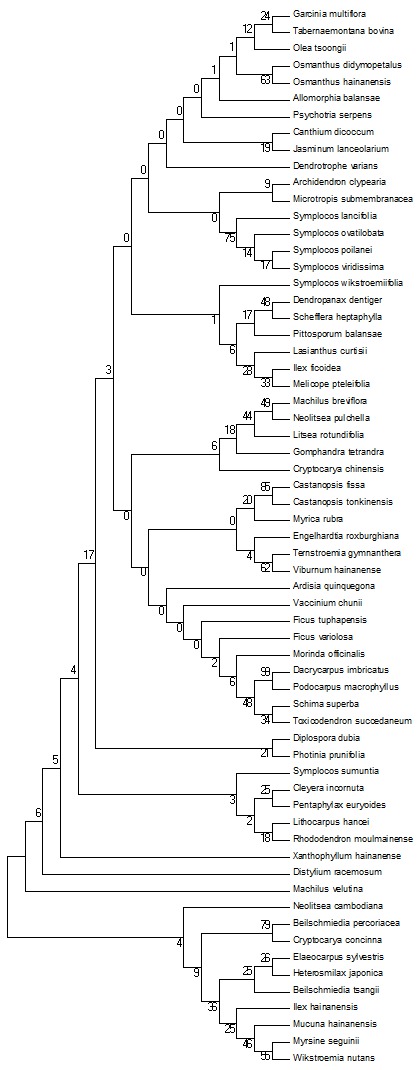


**Fig. S7 The phylogenetic tree of Bawangling tropical cloud forest using fragment combination of *trnH-psbA*+ITS**


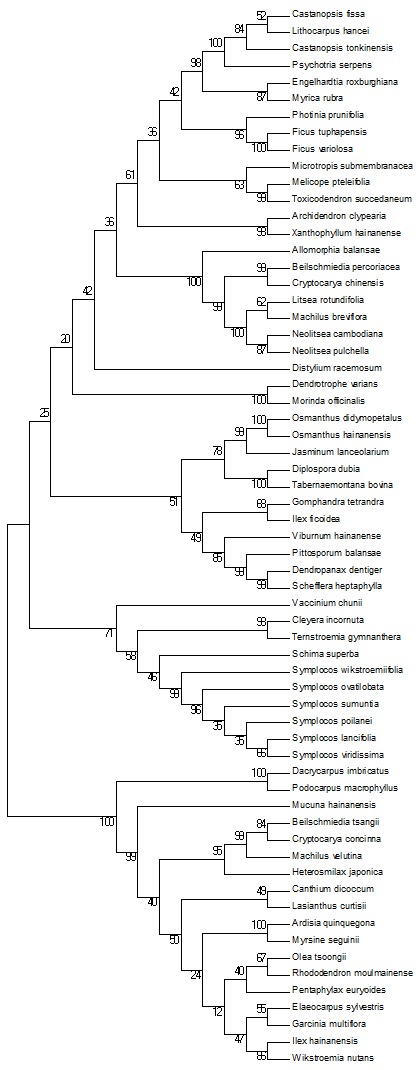


**Fig. S8 The phylogenetic tree of Bawangling tropical cloud forest using fragment combination of *rbcL+matK***


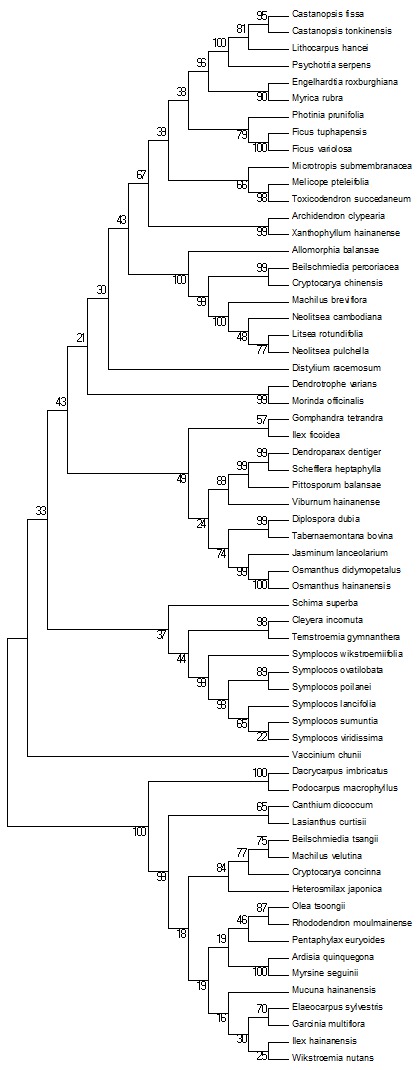


**Fig. S9 The phylogenetic tree of Bawangling tropical cloud forest using fragment combination of *matK+trnH-psbA***


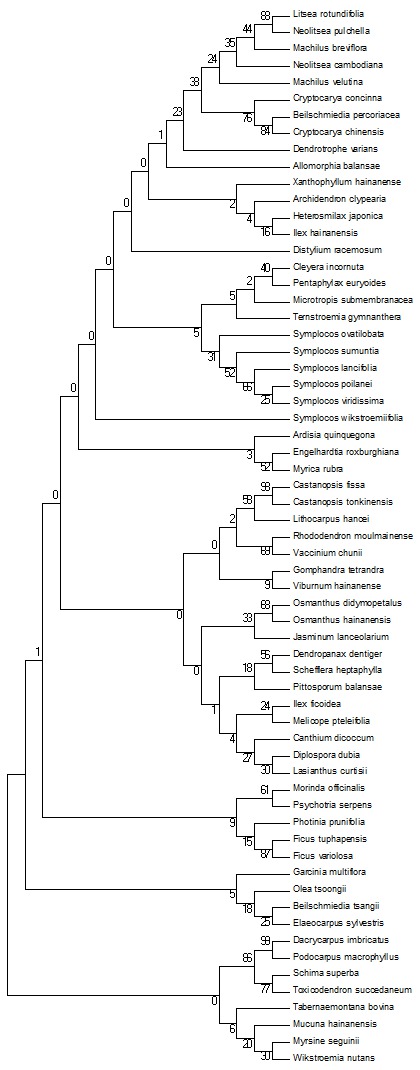


**Fig. S10 The phylogenetic tree of Bawangling tropical cloud forest using fragment combination of *rbcL+trnH-psbA***


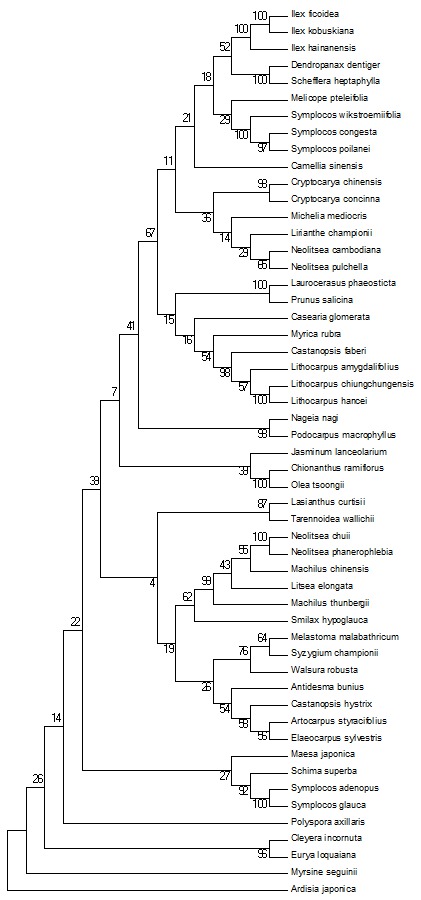


**Fig. S11 The phylogenetic tree of Limushan tropical cloud forest using fragment combination of *rbcL+matK+trnH-psbA+*ITS**


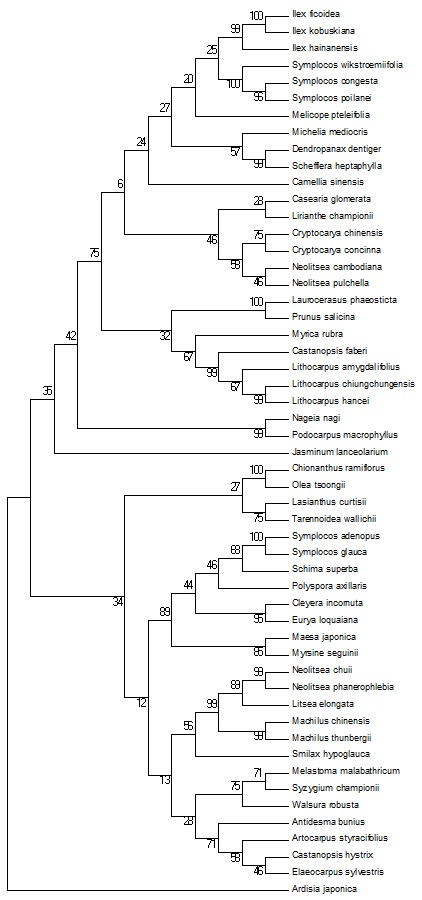


**Fig. S12 The phylogenetic tree of Limushan tropical cloud forest using fragment combination of *rbcL+matK+*ITS**


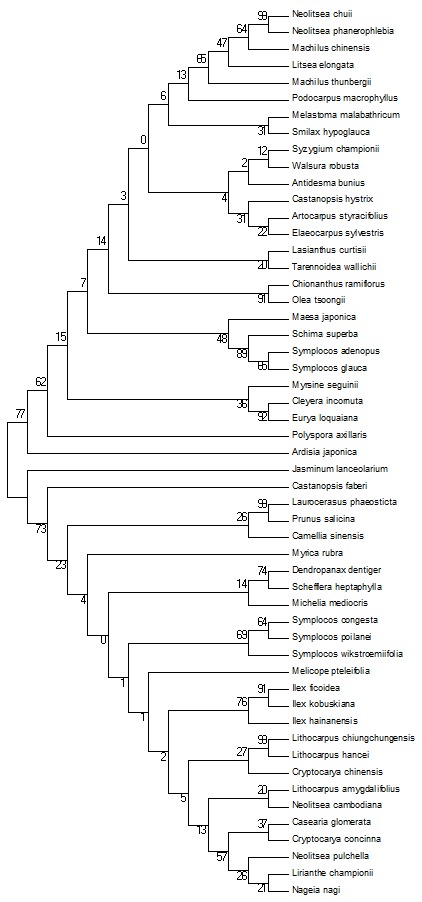


**Fig. S13 The phylogenetic tree of Limushan tropical cloud forest using fragment combination of *matK+trnH-psbA*+ITS**


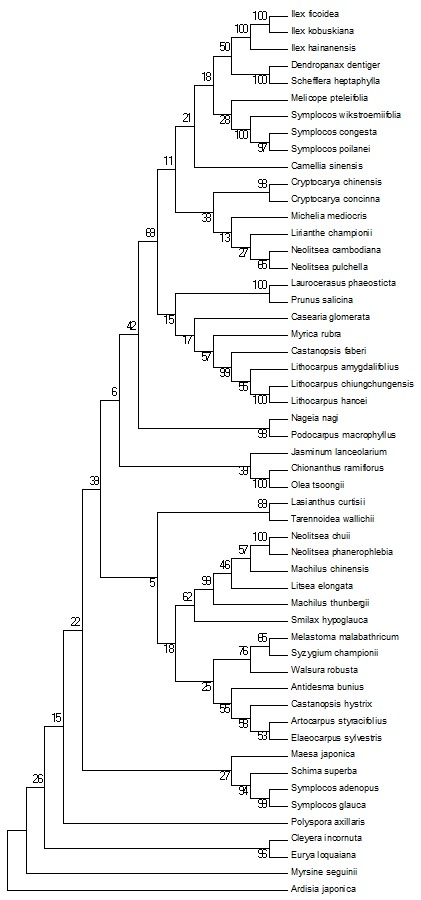


**Fig. S14 The phylogenetic tree of Limushan tropical cloud forest using fragment combination of *rbcL+trnH-psbA*+ITS**


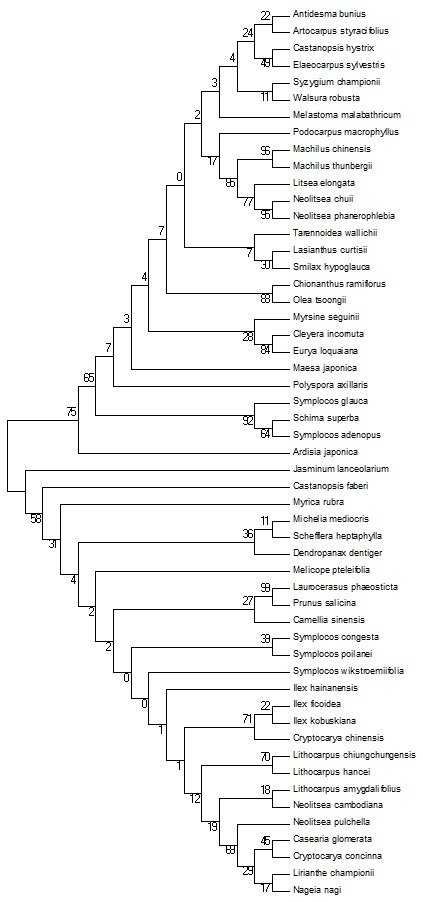


**Fig. S15 The phylogenetic tree of Limushan tropical cloud forest using fragment combination of *matK* +ITS**


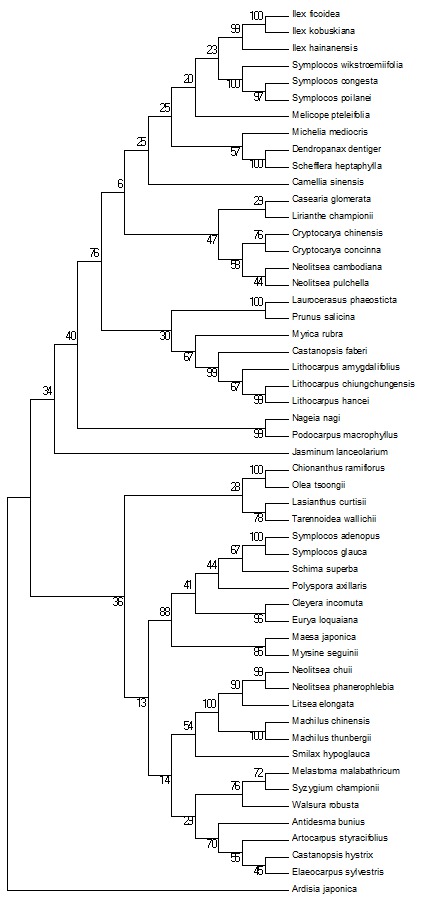


**Fig. S16 The phylogenetic tree of Limushan tropical cloud forest using fragment combination of *rbcL* +ITS**


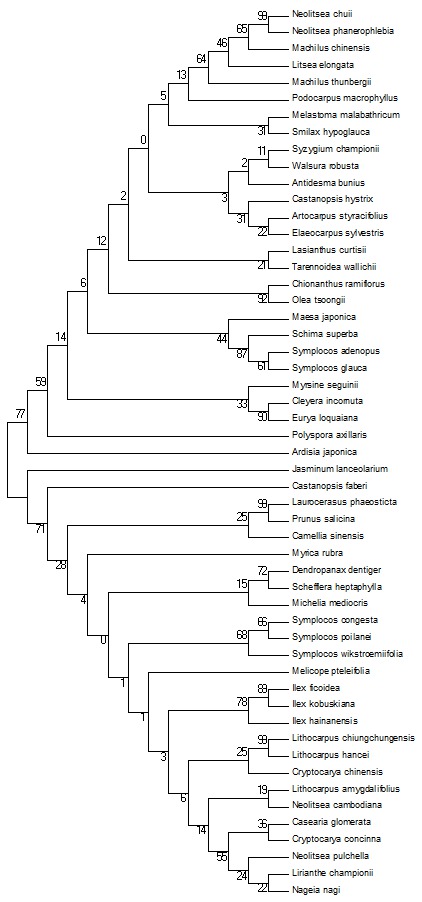


**Fig. S17 The phylogenetic tree of Limushan tropical cloud forest using fragment combination of *trnH-psbA*+ITS**


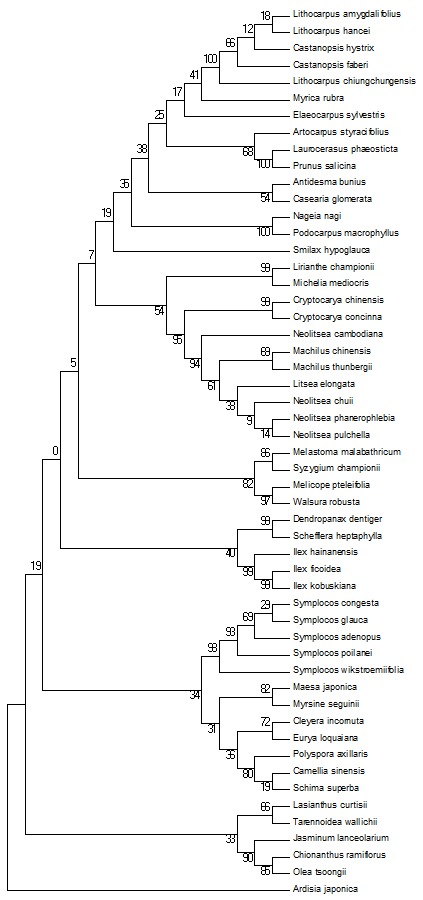


**Fig. S18 The phylogenetic tree of Limushan tropical cloud forest using fragment combination of *rbcL+matK***


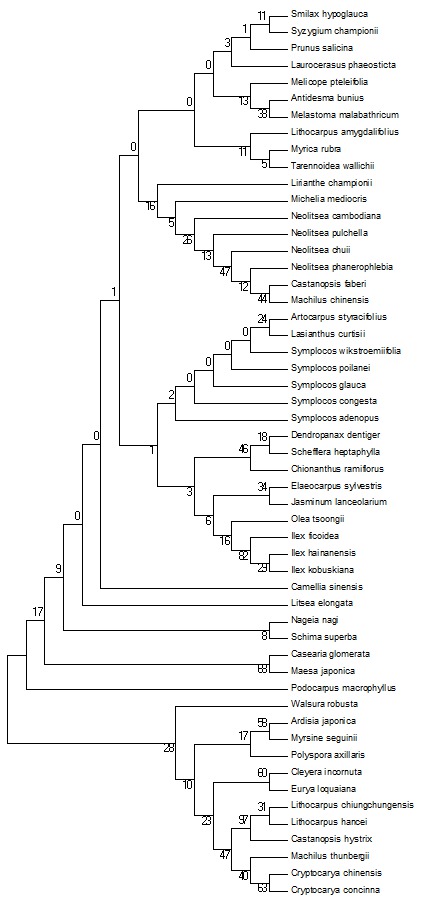


**Fig. S19 The phylogenetic tree of Limushan tropical cloud forest using fragment combination of *matK+trnH-psbA***


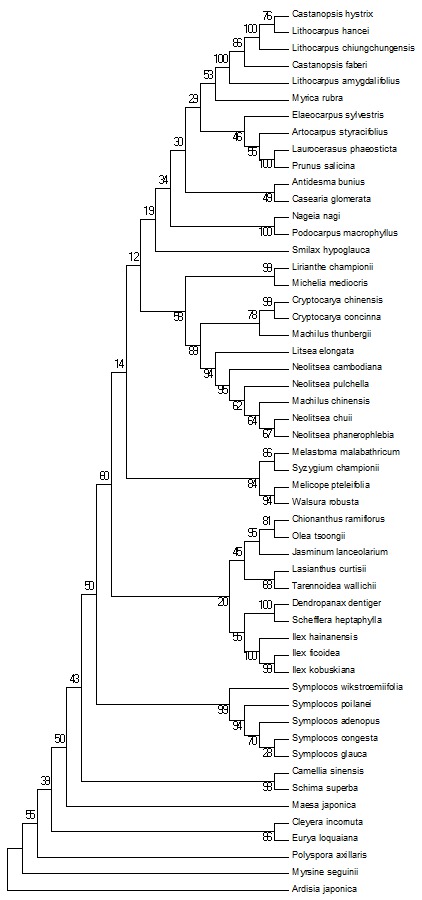


**Fig. S20 The phylogenetic tree of Limushan tropical cloud forest using fragment combination of *rbcL+trnH-psbA***


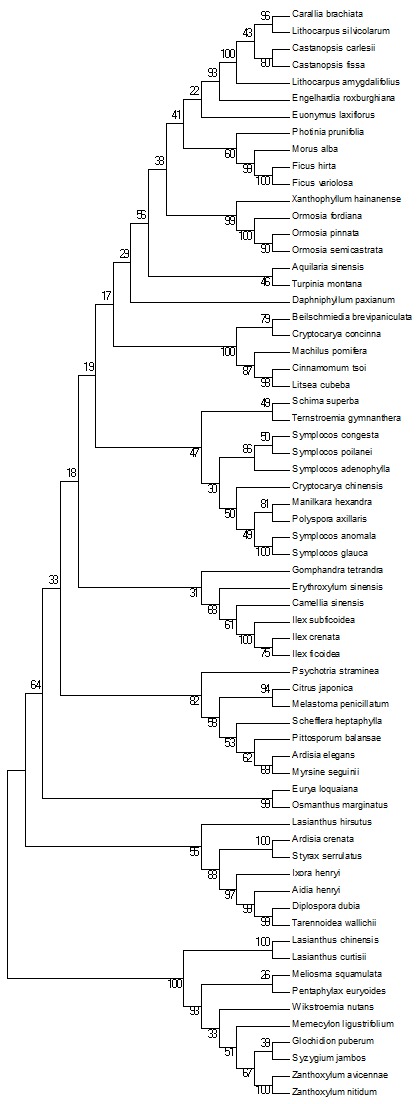


**Fig. S21 The phylogenetic tree of Jianfengling tropical cloud forest using fragment combination of *rbcL+matK+trnH-psbA*+ITS**


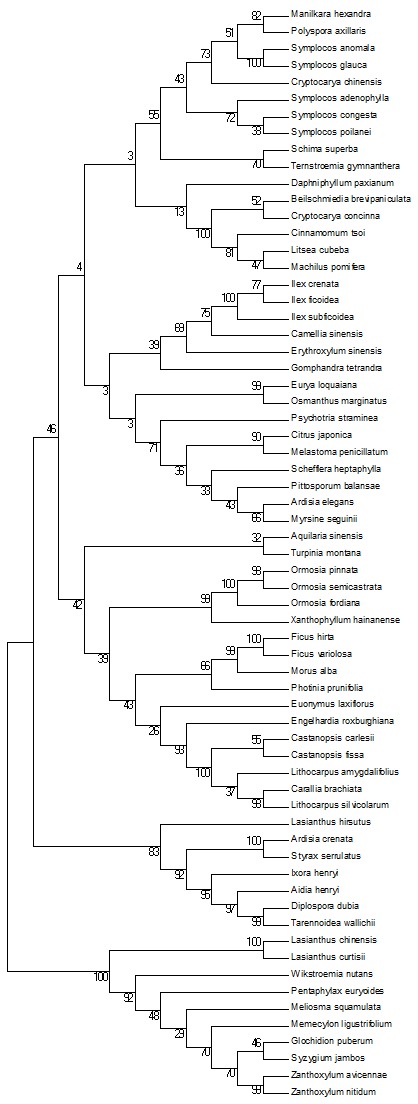


**Fig. S22 The phylogenetic tree of Jianfengling tropical cloud forest using fragment combination of *rbcL+matK+*ITS**


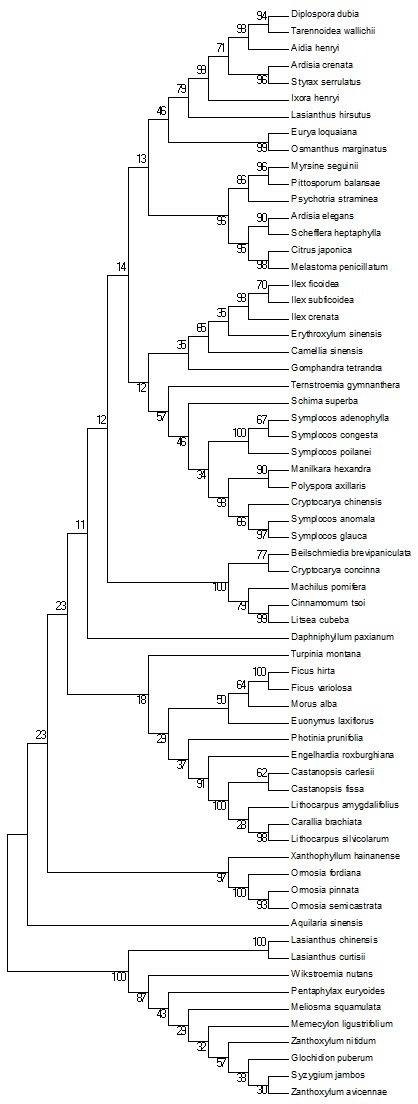


**Fig. S23 The phylogenetic tree of Jianfengling tropical cloud forest using fragment combination of *matK+trnH-psbA+*ITS**


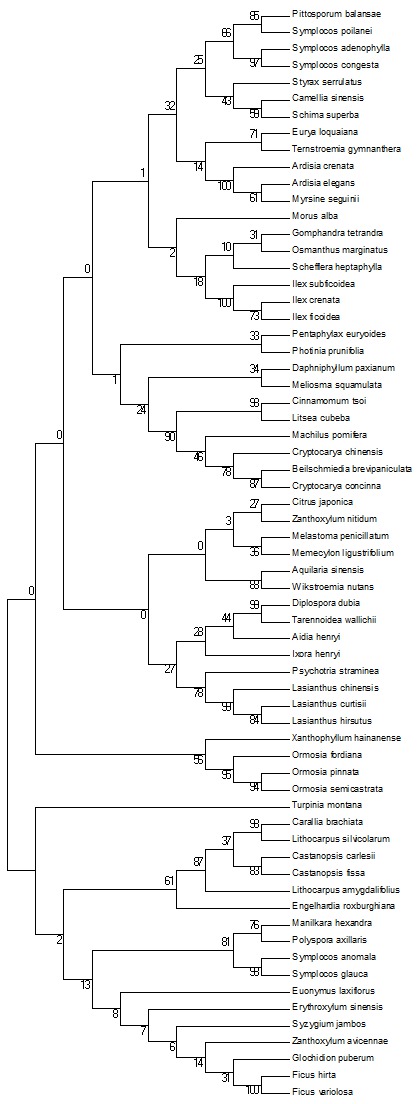


**Fig. S24 The phylogenetic tree of Jianfengling tropical cloud forest using fragment combination of *rbcL+trnH-psbA+*ITS**


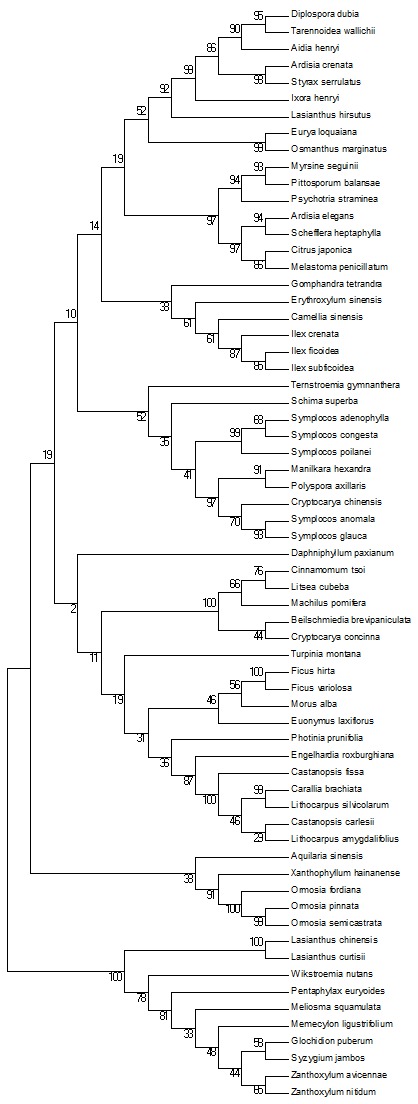


**Fig. S25 The phylogenetic tree of Jianfengling tropical cloud forest using fragment combination of *matK +*ITS**


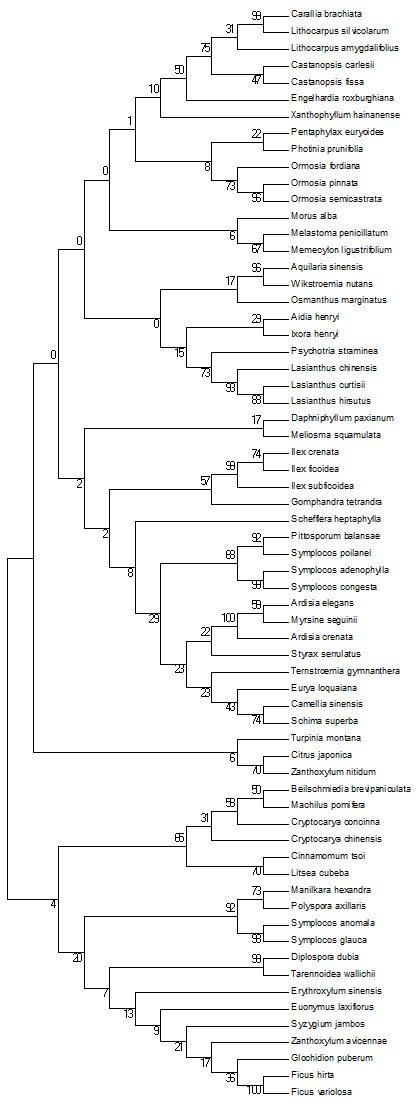


**Fig. S26 The phylogenetic tree of Jianfengling tropical cloud forest using fragment combination of *rbcL*+ITS**


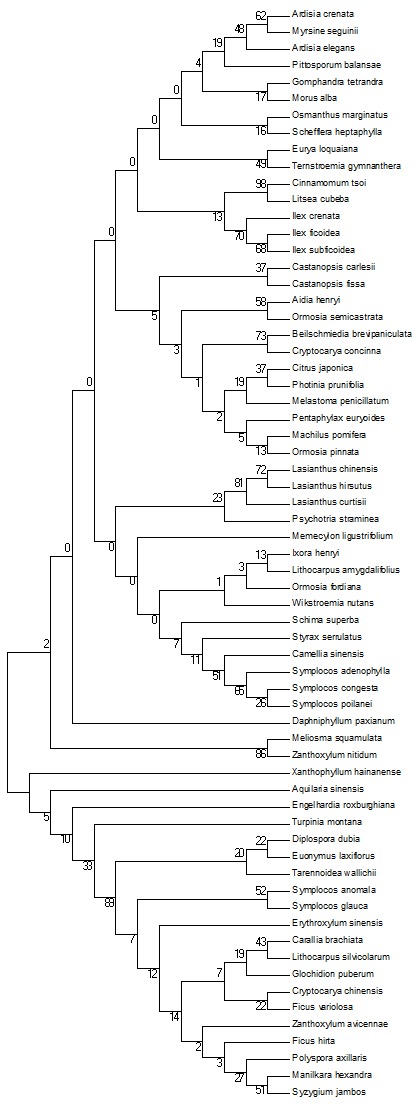


**Fig. S27 The phylogenetic tree of Jianfengling tropical cloud forest using fragment combination of *trnH-psbA*+ITS**


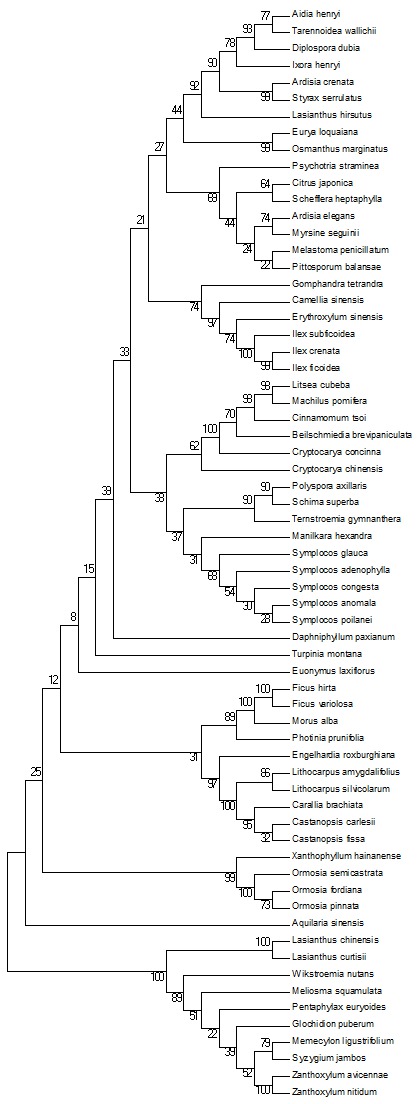


**Fig. S28 The phylogenetic tree of Jianfengling tropical cloud forest using fragment combination of *rbcL+matK***


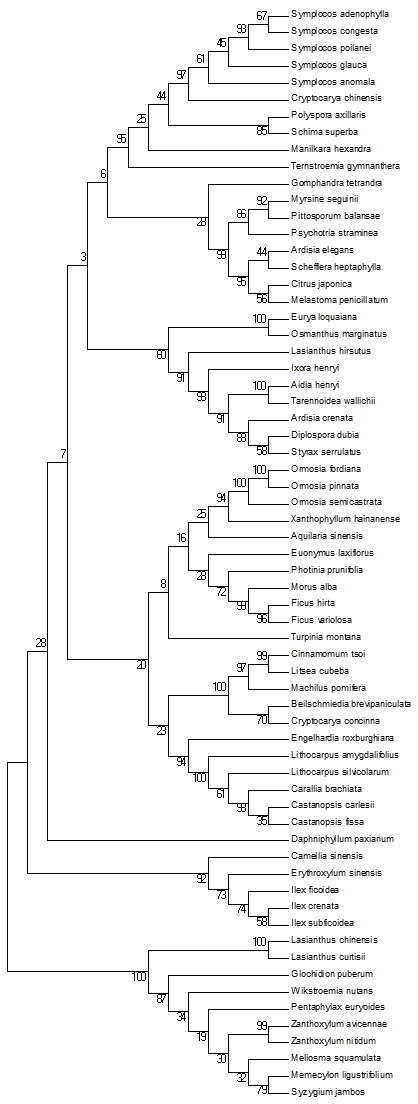


**Fig. S29 The phylogenetic tree of Jianfengling tropical cloud forest using fragment combination of *matK+trnH-psbA***


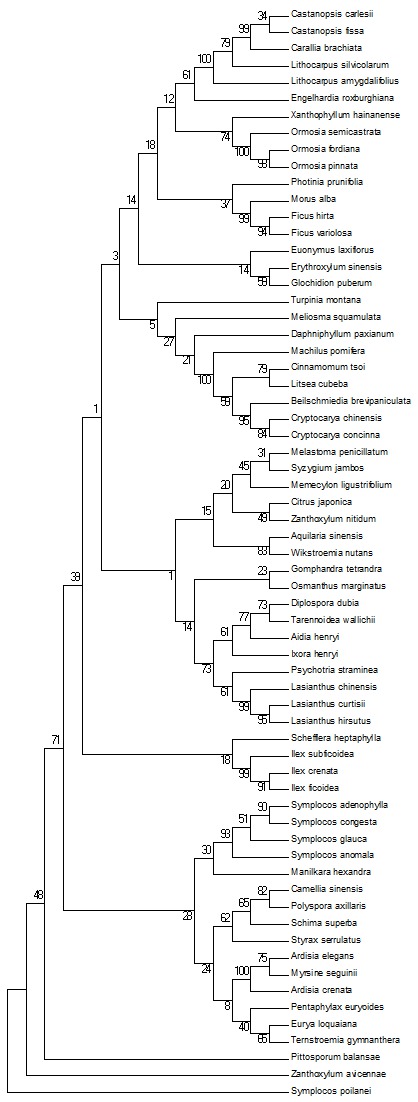


**Fig. S30 The phylogenetic tree of Jianfengling tropical cloud forest using fragment combination of *rbcL+trnH-psbA***
